# Progress in Fevers

**Published:** 1903-01-31

**Authors:** 


### PROGRESS IN FEVERS.

(*Concluded from page* 287.)

Scarlet Fever.—C. K. Millard[23] deals with the etiology of return cases of scarlet fever. By "return cases" is meant cases arising from contact with a patient discharged from hospital. The latter may be called the "infecting case." Six weeks is the very shortest time after discharge during which return cases are to be so regarded. An unhealthy condition of the nasal mucous membrane, with or without nasal or oral discharge, appears to be of great importance, whilst desquamation appears to be unimportant in determining the infectivity of a case. Boobbyer first pointed out in 1896 that the fatality of return cases is above the average, and Mason, Niven and others have confirmed this. Boobbyer blames the "old hospital cases," cases which have been detained many weeks in hospital, for spreading infection of a more virulent type. Return cases used to be attributed to premature discharge, a view shown by Niven and Simpson to be incorrect. On the contrary, it is now being doubted whether prolonged stay in hospital may not actually lead to return cases by interfering with elimination of infectivity, or by heaping up extraneous infection, to be carried out by the individual. Overcrowding, independently of shortened stay, appears to increase infectivity as shown by return cases. Two views concerning the origin and nature of the infection carried home by the infecting cases are dealt with. Niven and Simpson do not regard the patient as infective *per se*, but rather as having infection engrafted on him, the infective matter emanating from an acute case being breathed in by the convalescent in the ward and stored in his nostrils to be subsequently breathed out at home. The infectivity of an aural discharge may be derived from that stored up in the recesses of the nose by way of the Eustachian tube. Dr. Millard's own view is slightly different. He considers that the belief that the infection of scarlet fever is eliminated by the skin is supported by little evidence and is "rapidly waning." The lungs and air passages are more probably the chief channels of elimination, and hence elimination of infection is likely to be retarded in an atmosphere charged with infection breathed out by other patients, and that therefore the patient on leaving hospital is infectious, *per se*, and not merely "charged" with extraneous infection. The second view is regarded as a corollary of the first, both being probably true. The scarlet fever germ, finding a suitable nidus in the nasal mucous membrane, may persist for long, its activity being related to the presence of a discharge, "whether causally or consequentially is not yet certain." The practical conclusions drawn from these considerations are that aggregation is wrong in principle, that true isolation should be substituted, and that great attention should be paid to the disinfection of the nasal cavities. Experiments on these lines are now being carried out in accordance with suggestions made by a special committee of the Royal College of Physicians to whom Professor Simpson's report was submitted by the Metropolitan Asylums Board. Similar attempts at disinfection with a fortnight's isolation only in special hospital wards, as carried out by Dr. Niven in Manchester, have, so far, led to no diminution of return cases, but the isolation period should be increased to at least four, and preferably to six, weeks. Dr. Millard's[24] conclusion is that "the hospital isolation of scarlet fever, regarded as a preventive measure, has been a failure." E. D. Marriott[25] considers, having regard to the number and gravity of return cases, that "the aggregation of the infective sick lacks the sanction of both science and experience." W. H. Line[26] thinks the profession owes a debt of gratitude to Dr. Millard for his painstaking and courageous investigation of the effects of hospitalism on the spread of scarlatina, although he admits case mortality and complications have been reduced, and the type of fever appears milder than in the pre-hospital days. S. Cameron[27] considers that hospital isolation will continue necessary for the working and lower middle classes where home isolation is impossible. It is suggested that school authorities would in self-defence have to provide isolation if municipal authorities did not, so serious would be the interruptions to education in the absence of hospitals. Overcrowding in isolation hospitals is to be deprecated, and there should be also isolation for doubtful cases, whilst acute and convalescent cases should be separated. R. de Bovis[28] reviews the history of traumatic scarlatina. It is 40 years since English authors first directed attention to the occurrence of scarlatiniform eruptions in the subjects of operation or other wounds, the history commencing with the publication of writings by Sir James Paget and G. May in 1864. Since then the subject has from time to time been brought forward by Goodhart and Howse, in this country, and by Verneuil, Tremblay, Hoffa, Koch and others elsewhere. Hoffa classed many recorded cases as erythematous, drug, or transfusion rashes, or as other infectious rashes, septicæmic, diphtheritic, etc., and only admitted six or seven cases as authentic. De Bovis has collected nearly 150 cases, of which only about 100 are accompanied by fairly full notes. Two-thirds of the cases were in males, and nearly 80 per cent. occurred in patients under fifteen. No special traumatism was concerned, septic and aseptic cases were alike noted. In over 50 per cent. less than three days intervened between the traumatism and the eruption; in 13 cases it was upwards of four weeks. Initial symptoms included fever, chill, gastro-intestinal disturbance, and sore throat, the last-named being milder than is usual in medical cases. The rash generally appeared within 24 hours, sometimes starting as a red ring round the wound, with red bands radiating to adjacent parts. Healing of the wound was generally retarded. Albuminuria was noted 16 times. The mortality was 9 out of 125 cases. Recrudescence of the rash two or three times occurred in two cases. C. H. Powers[29] also mentions a medical case of scarlet fever in which the rash and other symptoms reappeared 14 days after the beginning of the illness. V. C. de Boinville[30] reports a fatal case in which epistaxis and petechiæ occurred 15 or 16 days after the onset of the disease during apparently normal convalescence and desquamation. A case of purpura fulminans is also reported by H. Biss.[31] The patient, a boy 3 years old, was convalescing fairly satisfactorily from a very severe attack of scarlet fever. On the eighteenth or nineteenth day extensive subcutaneous hæmorrhages appeared, followed by hæmatemesis, rectal hæmorrhages, and death in 36 hours. Post-mortem the kidneys were found almost entirely converted into fat.

[23] Brit. Med. Jour., Aug. 16. [24] Ibid., Sept. 27, p. 1000. [25] Ibid. [26] Midland Med. Jour., Sept. [27] Brit. Med. Jour., Nov. 1, p. 1450. [28] Med. Press and Circular, Sept. 24. [29] Lancet, Aug. 16, p. 480. [30] Ibid., Aug. 9, p. 359. [31] Ibid., Aug. 2.

